# A Study of *Burkholderia pseudomallei* in the Environment of Farms in Thanlyin and Hmawbi Townships, Myanmar

**DOI:** 10.4269/ajtmh.18-0678

**Published:** 2019-02-18

**Authors:** Mo Mo Win, Thaung Hla, Khin Phyu Phyu, Wah Wah Aung, Kyi Kyi Nyein Win, Su Nyein Aye, Thin Thin Wah, Khin Mar Aye, Tin Tin Htwe, May Than Htay, Kyaw Kyaw San, David Allan Brett Dance

**Affiliations:** 1 Department of Medical Research, Yangon, Myanmar;; 2 Lao-Oxford-Mahosot Hospital-Wellcome Trust Research Unit, Microbiology Laboratory, Mahosot Hospital, Vientiane, Lao PDR;; 3 Centre for Tropical Medicine and Global Health, University of Oxford, Oxford, United Kingdom;; 4 Faculty of Infectious and Tropical Diseases, London School of Hygiene and Tropical Medicine, London, United Kingdom

## Abstract

Melioidosis is a tropical infection, first described in Myanmar but now rarely diagnosed there, which is widespread in Southeast Asia. The infection is predominantly acquired by people and animals through contact with soil or water. This study aimed to detect the causative organism, *Burkholderia pseudomallei*, in environmental samples from farms in Thanlyin and Hmawbi townships near Yangon, Myanmar. One hundred and twenty soil samples and 12 water samples were collected and processed using standard microbiological methods. *Burkholderia* species were isolated from 50 of the 120 (42%) soil samples but none of the water samples. Arabinose assimilation was tested to differentiate between *B. pseudomallei* and the nonpathogenic *Burkholderia thailandensis*, and seven of 50 isolates (14%) were negative. These were all confirmed as *B. pseudomallei* by a species-specific multiplex polymerase chain reaction (PCR). This is the first study to detect environmental *B. pseudomallei* in Myanmar and confirms that melioidosis is still endemic in the Yangon area.

*Burkholderia pseudomallei* is a small, motile, Gram-negative, non-fermentative bacillus which causes melioidosis in both humans and animals. It was first described in Myanmar by Whitmore and Krishnaswami in 1911 in a 40-year-old morphine addict who died from acute fulminating peritonitis with subcutaneous thigh abscesses.^1^ *Burkholderia pseudomallei* is an environmental saprophyte found in soil and water in the tropics, and infection is usually thought to be acquired through inoculation or inhalation from soil or water containing the organism. People with occupations such as farming or gardening in contaminated environments have a high risk of acquiring *B. pseudomallei*, especially if they have underlying conditions that predispose them to infection such as diabetes mellitus.^2^

Although melioidosis was said to be found in about one in every 20 autopsies performed in Rangoon General Hospital in 1917,^3^ melioidosis is little known and rarely diagnosed in Myanmar these days.^4^ *Burkholderia pseudomallei* can readily be isolated from soil and surface water, and the detection of the organism in the environment can help identify areas of risk. We, therefore, undertook a study to investigate the presence of *B. pseudomallei* in agricultural lands in two townships on the outskirts of Yangon.

Thanlyin and Hmawbi townships were selected for sampling on the basis of finding cases of culture-positive melioidosis from these areas at Yangon General Hospital in 2009.^5^ Two farms were selected near the center of each township, plus one farm each in an easterly, westerly, northerly, and southerly direction, respectively. Their locations are shown in Figure 1.

**Figure 1.**
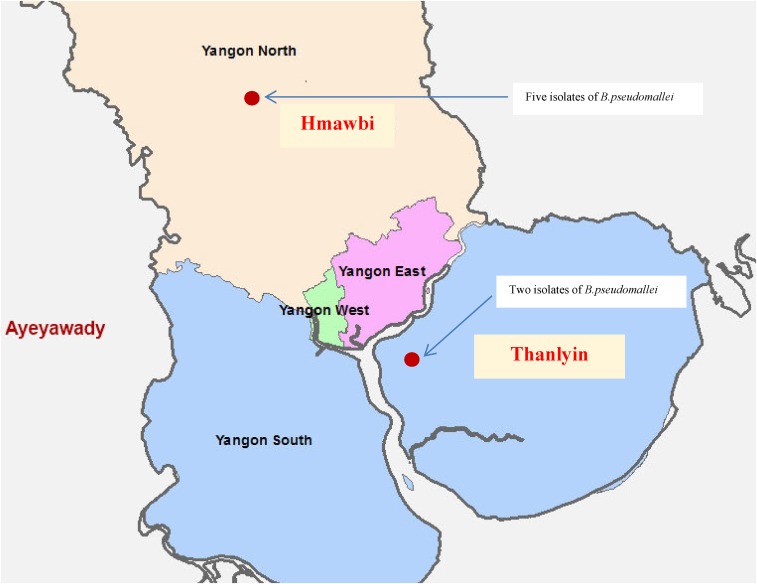
Map showing the locations of the farms where *Burkholderia pseudomallei* was isolated. This figure appears in color at www.ajtmh.org.

A total of 120 soil and 12 water samples were collected from the six farms in each township (10 soil samples and one water sample from each farm). Soil was collected from the farmland and water from that used for irrigation of the farms. At each site, a hole was dug to a depth of approximately 30 cm using a spade, and 100 g of soil was collected from the base of the hole using a trowel, which was washed and wiped before sampling the next hole, placed in a sterile plastic bag, and labeled. Water samples (100 mL) were collected from each site and placed into sterile containers. All samples were transported to the laboratory at ambient temperature.

The samples were processed using the method of Thomas et al.^6^ A total of 50 grams of each soil sample was transferred to a sterile glass jar and mixed with 100 mL of distilled water. After vigorous shaking, the mixture was allowed to stand overnight at ambient temperature. Eighty milliliters of supernatant was centrifuged at approximately 1,000 g for 20 minutes and the deposit was collected by using a sterile swab and inoculated into 100 mL of MacConkey broth (containing 0.001 percent of crystal violet, 25 units/mL of penicillin G, and 50 units/mL of streptomycin sulfate) and incubated at 37°C for 48 hours. Water samples were filtered through 0.45 μm filters (Millipore, Merck, Life Sciences, Burlington, MA). The filter paper was incubated in MacConkey broth (as previously) at 37°C for 48 hours.

Following incubation, the MacConkey broths were subcultured on MacConkey agar (for other *Burkholderia* species) and Ashdown agar at 37°C for 48 hours. Colonies with morphology suggestive of *Burkholderia* species on MacConkey agar or *B. pseudomallei* on Ashdown’s agar were presumptively identified using Gram staining and biochemical tests (positive arginine dihydrolase, negative lysine and ornithine decarboxylase reactions, negative *ortho*-Nitrophenyl-β-galactoside (ONPG), and positive nitrate reduction tests).^7^

Isolates of presumptive *Burkholderia* species were tested for L-arabinose assimilation to distinguish the pathogenic *B. pseudomallei* (negative) from the nonpathogenic *Burkholderia thailandensis* and other positive species. This was carried out by using the method of Wuthiekanun et al.^8^ The agar consisted of minimal salts solution (25 mL), 2% agar solution (Himedia Laboratories Pvt. Ltd., Mumbai, India) (75 mL), and 10% carbohydrate (L-arabinose or glucose; Sigma -Aldrich Co., St. Louis, MO) solution (2 mL). Each solution was sterilized separately before mixing and pouring in 150 × 15-mm petri dishes. The agar plates were spotted with 3 µL of an overnight bacterial culture suspended in normal saline, diluted to turbidity equivalent to a 0.5 McFarland standard. The plates were incubated at 37°C for 48 hours and the presence or absence of growth on L-arabinose was recorded. The glucose agar was used as a growth control and a known *B. pseudomallei* culture as an arabinose-negative control.

Presumptive *Burkholderia* species were identified using a multiplex PCR developed by Kerdsin et al.^9^ The assay differentiates *B. pseudomallei*, *B. thailandensis*, *Burkholderia mallei*, and *Burkholderia ubonensis* on the basis of their type III secretion system cluster genes and *Burkholderia cepacia* complex by their recA genes.

Of the 120 soil samples tested, presumptive *Burkholderia* species were isolated from 50 samples (31 from Hmawbi Township and 19 from Thanlyin Township). There were no isolates obtained from the 12 water samples. The arabinose assimilation test was negative in 7/50 isolates and those isolates were regarded as presumptive *B. pseudomallei*. The remaining 43 isolates (86%) were arabinose positive and, thus, considered nonpathogenic. The multiplex PCR was performed on the seven arabinose-negative isolates and 32 representative arabinose-positive isolates. All seven arabinose-negative isolates were confirmed as *B. pseudomallei.* Five came from two of the six farms in Hmawbi Township and two from one of the six farms in Thanlyin Township.

Melioidosis is a severe disease which requires specific and prolonged treatment to prevent death and relapse. However, difficulties in laboratory diagnosis of melioidosis may mean that optimal treatment is never given or delayed, resulting in mortality that may be as high as 70–80%.^10^

In this study, we identified seven isolates of *B. pseudomallei* from both Hmawbi and Thanlyin townships. This is the first time the organism has been isolated from soil within Myanmar, confirming that *B. pseudomallei* is still present in the environment in and around Yangon despite the dearth of cases of melioidosis reported there in recent years. It is, thus, likely that melioidosis continues to occur but is not currently being diagnosed in Myanmar because of relatively underdeveloped diagnostic microbiology services in many hospitals and a lack of awareness of melioidosis and *B. pseudomallei* among doctors and laboratory staff.

The detection of *B. pseudomallei* in environmental samples is notoriously difficult, and the methods used in this study may actually have underestimated the number of sites at which *B. pseudomallei* was present because we did not use the international consensus method for soil culture^11^ or PCR following enrichment culture, which has been shown to have greater sensitivity than culture in other studies.^12^ The findings of this small study are only preliminary. Nonetheless, this study undoubtedly confirms that agricultural workers in the Yangon area are at risk of exposure to *B. pseudomallei* and are, thus, at risk of melioidosis, especially if they have predisposing conditions such as diabetes mellitus. Further study of environmental *B. pseudomallei* in Myanmar is indicated to construct risk maps and identify places in which enhanced surveillance for melioidosis should be undertaken.
